# Gastrointestinal endoscopy satisfaction questionnaire is a valid tool to measure patient satisfaction in Asian country

**DOI:** 10.1097/MD.0000000000011477

**Published:** 2018-07-20

**Authors:** Jin Young Yoon, Jae Myung Cha, Min Seob Kwak, Jung Won Jeon, Hyun Phil Shin, Kwang Ro Joo, Joung Il Lee

**Affiliations:** Department of Internal Medicine, Kyung Hee University Hospital at Gangdong, Kyung Hee University School of Medicine, Seoul, Korea.

**Keywords:** gastrointestinal endoscopy, patient satisfaction, quality, translation, validation

## Abstract

Patient satisfaction is a key quality indicator of gastrointestinal endoscopy (GIE). The gastrointestinal endoscopy satisfaction questionnaire (GESQ) was recently developed to assess patient satisfaction undergoing GIE in Europe; however, it was not validated in Asian countries. We aimed to translate and validate the GESQ in Korea and identify predictors for patient satisfaction during GIE.

Translation of the original GESQ was performed according to accepted linguistic validation guidelines. Between March 2016 and July 2016, 350 consecutive patients were asked to complete a GESQ after GIE at Kyung Hee University Hospital. Total sum of scores was transformed from 0 to 100 by the formula: (Score-lowest possible/Score range) × 100.

Exploratory and confirmatory factor analyses for construct validation reconfirmed that 4 factors were extracted from the Korean GESQ. Internal consistency reliability was acceptable with an overall Cronbach α score of 0.87. Female and nonsmoker were associated with less satisfaction with GIE (*P* = .021 and .006, respectively). Other factors, including age, alcohol, education or economic level, sedative endoscopy, gastroscopy with or without colonoscopy, experience of previous endoscopy, and additional examinations such as biopsy, were not associated with patient satisfaction during GIE.

The Korean version of the GESQ was a valid and acceptable tool to measure satisfaction in patients who had undergone a GIE in Korea. Patient satisfaction measurement could contribute to systematic improvement of qualified GIE.

## 1 Introduction

Gastrointestinal endoscopy (GIE), such as esophagogastroduodenoscopy (EGD) and colonoscopy, is the most effective method for reducing gastric cancer and colorectal cancer-associated mortality.^[1,2]^ For a population-based endoscopy screening program, quality assurance of GIE must be carefully considered. In the past decade, quality indicators for GIE have shifted from the view of the health care provider to the view of patient experience, including patient satisfaction.^[3]^ Satisfied patients are more likely to be compliant with repeat attendance and participation in population-based screening programs.^[4]^ Patient satisfaction based on their cognition and experiences became an important quality indicator for GIE. Therefore, measurement of patient satisfaction for GIE is an important element to improve the quality of GIE. In Asian countries, there have been few validated tools to measure patient satisfaction during GIE,^[5]^ and it is unclear which predictors may affect patient satisfaction during GIE. Previous measurement tools to evaluate patient satisfaction were limited as they lacked evaluation of essential factors for patient satisfaction, such as care systems after GIE.^[6,7]^

Recently, the gastrointestinal endoscopy satisfaction questionnaire (GESQ) was developed in Europe and includes 21 questions and is categorized into 4 domains including information before endoscopy, skills and hospital, pain or discomfort during or after endoscopy, and information after endoscopy. Most questionnaires used for patient satisfaction during GIE were developed in Western countries and were not validated in Asian countries.^[8,9]^ The GESQ was also not validated in Asian countries including Korea. The GESQ should be validated in each country after translation as health care utilization patterns and resources are different between Asian and Western countries.

The purpose of this study was to validate the Korean version of the GESQ (K-GESQ) to measure patient satisfaction and identify predictors for patient satisfaction with GIE.

## 2 Materials and methods

### 2.1 Patients

We enrolled consecutive patients aged at least 18 years, who had undergone EGD or colonoscopy at Kyung Hee University Hospital between March 2016 and July 2016. Patients were asked to complete the K-GESQ. Patients were reassured that their responses would be anonymous and confidential. Patients were also asked to complete another questionnaire gathering information on the following variables: baseline demographic characteristics including age, gender, and smoking/alcohol habits; social status including educational status and income levels; type of endoscopy (EGD, colonoscopy, or both); previous endoscopy experience; and additional costs due to the need for biopsy forceps, test for *Helicobacter pylori*, or immunohistochemical staining after endoscopic examination. These variables were analyzed to identify predictors of patient satisfaction during GIE. Patients who underwent in-hospital or emergency GIE, such as endoscopic interventions for acute gastrointestinal bleeding or obstruction, were excluded from this study. The study was approved by the Institutional Review Board of Kyung Hee University Hospital (KHNMC IRB 2016-01-002), and all patients provided written consent for this study.

### 2.2 Translation of GESQ to Korean

After getting permission from Elsevier and the original corresponding author (Hutching HA),^[6]^ the English version of the GESQ for measuring patient satisfaction during GIE was translated to Korean. For the English-Korean translation, the forward and back translation method was used.^[10]^ One professional translator, who was a native speaker of Korean and fluent in English, produced a K-GESQ, then another who was a native speaker of English and fluent in Korean translated the K-GESQ back into English. When discrepancies occurred between the original and back-translated versions, we assessed the significance of these discrepancies and modified the translated version to a more appropriate and adequate translation.

We converted the negative status of all component items to 1 and positives to 5 for analyzing and validating the GESQ. Three-point Likert scales (1, 3, or 5) and binary questions (1 or 5) were applied by same rule. Total sum of scores was transformed from 0 to 100 by the formula: (Score-lowest possible/Score range) × 100.^[6]^

### 2.3 Validation of the K-GESQ

Content validity of the K-GESQ was determined for the areas measured by each test item. A correlation matrix was calculated to identify redundant or irrelevant items. If the correlation coefficient between 2 items was not significant via Pearson correlation coefficients, the items were eliminated.^[11]^ Bartlett test of sphericity and the Kaiser-Mayer-Olkin (KMO) measure were used to assess the suitability of factor analysis. Factor analysis was computed for evaluating the degree each item contributed to the total of the satisfaction spectrum using principal component analysis with direct oblimin rotation. Factors were extracted if the eigenvalue was >1, and we considered the criterion for contribution to be achieved if factor loading was ≥0.4.^[12]^

Structural validity of the K-GESQ was demonstrated with confirmatory factory analysis (CFA). The acceptable criteria for the CFA model based on multiple fit indices were as follows: χ^2^/d*f* < 3 is good and <5 is sometimes permissible, comparative fit index (CFI) > 0.95 is great, >0.9 traditional, and >0.8 sometimes permissible, root-mean-square error of approximation (RMSEA) < 0.05 is good, 0.05 to 0.1 moderate, and >0.1 bad, standardized root mean square residual (SRMR) < 0.09 is good, and goodness-of-fit index (GFI) > 0.95 is good and 0.90 acceptable.^[13–15]^

Construct validity of the K-GESQ was assessed through convergent and discriminant validity. To assess the convergent validity of the K-GESQ, it must correlate with previous existing scales. Unfortunately, tools measuring patient satisfaction with GIE have not been developed or established in Korea. Therefore, we used a 5-point Likert scale (very satisfied-satisfied-neutral-dissatisfied-very dissatisfied) to assess the convergent validity of the K-GESQ. The Pearson correlation coefficient was measured between 1 and 0 (1 indicated the K-GESQ was very similar to a 5-point Likert scale, 0 indicated the K-GESQ was not related to a 5-point Likert scale and an entirely different calibration, and values near 0.5 indicated that the K-GESQ was suitable for developing into a questionnaire better than a 5-point Likert scale).^[16]^ Discriminant validity between subscales was verified with a relatively low correlation using Pearson correlation coefficient.

Internal consistency for verifying reliability of the GESQ was tested by calculating corrected item-total correlations; items were regarded as acceptable if corrected item-total correlations were below 0.2 (little relation) or above 0.8 (too high relation), based on the criteria applied to the original GESQ.^[6,17]^ Cronbach α, which determines the degree to which each subscale measures a single construct, was acceptable for internal consistency when it was 0.70–0.95.^[12]^ Reproducibility as test–retest reliability was not checked, because the patients would have to undergo repeat endoscopy by the same endoscopist under the same circumferences at another time.

### 2.4 Statistics

As the required sample size for assessing questionnaire validity was at least 7 times the number of items,^[3]^ more than 160 patients needed to complete the K-GESQ with 22 items. Continuous variables were presented as mean and standard deviations (SDs) and were compared using 2-sample *t* tests. Categorical variables were presented as numbers and percentages and compared using Chi-squared or Fisher exact tests. Pearson correlation coefficient was also used to examine relationships between variables. Statistical significance was noted at a 2-tailed *P*-value <.05. Statistical analyses were performed using the statistical software package SPSS 18.0 for Windows (SPSS Inc, Chicago, IL) and AMOS 5.0 programs (Arbuckle, 2003).

## 3 Results

### 3.1 Patients

Baseline information from 350 participants is summarized in Table 1. In total, 56.6% were female (N = 198), and the mean patient age was 53.6 ± 13.4 years. Indications for endoscopy were screening (39.8%), surveillance (16.9%), nonspecific gastrointestinal or alarm symptoms (36.4%), and abnormal findings at other hospitals (3.4%). Participants underwent EGD only (32.0%), colonoscopy only (12.3%), or both EGD and colonoscopy (55.7%). The majority (94.3%) of participants underwent endoscopy under sedation, and approximately 87.1% of participants had previous experience with endoscopy.

**Table 1:**
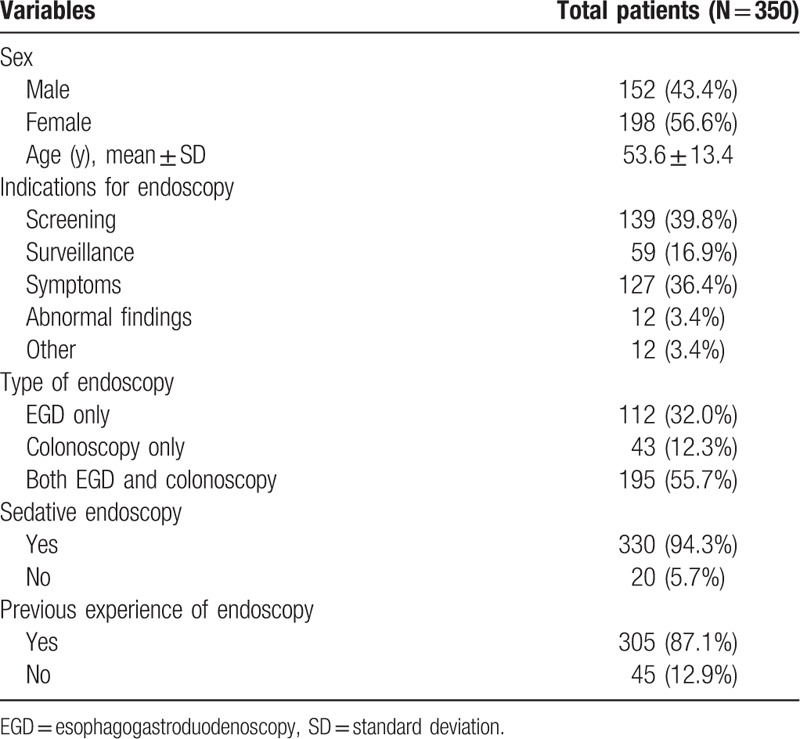
Baseline characteristics of the study population.

### 3.2 Missing values and correlation matrix

The score for each item ranged from 3.95 ± 0.85 to 4.77 ± 0.93. The rate of missing values for each item ranged from 0% to 1.4%, indicating that the K-GESQ was acceptable and interpretable. The missing values were fulfilled based on expectation–maximization. In the 21 × 21 correlation matrix, all items were significantly correlated with the other items in the same subscale, and no item had to be eliminated.

### 3.3 Validation of the K-GESQ

The result of Bartlett test of sphericity was significant (χ^2^ = 2888.30, *P* < .001), and the value of KMO was 0.874, implying that these data were suitable for factor analysis. According to exploratory factor analysis (EFA), all 21 items could be meaningfully clustered into 4 factors of information before endoscopy, skills and hospital, pain or discomfort during or after endoscopy, and information after endoscopy. Table 2 shows the factor loadings, namely the correlations between each individual item and the subscale to which it belonged. The 4 factor categories accounted for 57.81% of the total variance and were evenly distributed across the factors. CFA was conducted to determine acceptability of the extracted 4-factor model to the K-GESQ data. The value of χ^2^/d*f* was 2.1 (good), CFA was 0.938 (traditional permissible), RMSEA was 0.058 (95% confidence interval, 0.048–0.067) (regarded as moderate), SRMR was 0.054 (good), and GFI was 0.916 (acceptable). Therefore, the K-GESQ structure reached the criterion cut-off and was acceptable.

**Table 2:**
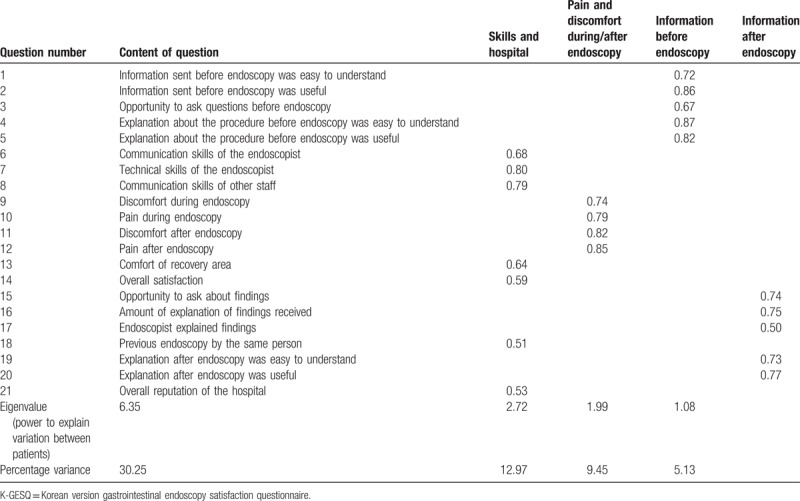
Exploratory factor analysis of K-GESQ.

Table 3 shows the inter-correlations between the K-GESQ domains. Pearson correlation coefficients between domains were all comparatively low (<0.70) and revealed that the 4 subscales consisting of 21 items were not collinear, suggesting separate satisfaction scales. The correlation coefficient between the K-GESQ and 5-point Likert satisfaction scale to assess convergent validity was 0.513 (*P* < .001). Thus, the K-GESQ demonstrated an acceptable level of convergent validity. For internal consistency reliability, the Cronbach α for each subcategory ranged from 0.72 to 0.82, which met the threshold criterion range of 0.70 to 0.95 (Table 4). The overall Cronbach α score for the K-GESQ was 0.87, and the α values of the subcomponents were as follows: skills and hospital component (0.77), pain or discomfort component during or after endoscopy (0.81), information component before endoscopy (0.82), and information component after endoscopy (0.72). These results showed that all components of the K-GESQ had favorable to high internal consistency. All corrected item-component correlations were between 0.34 and 0.75 and were acceptable.

**Table 3:**
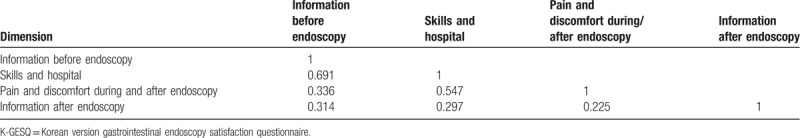
Correlations between the dimensions of the four factors of the 21-item K-GESQ.

**Table 4:**
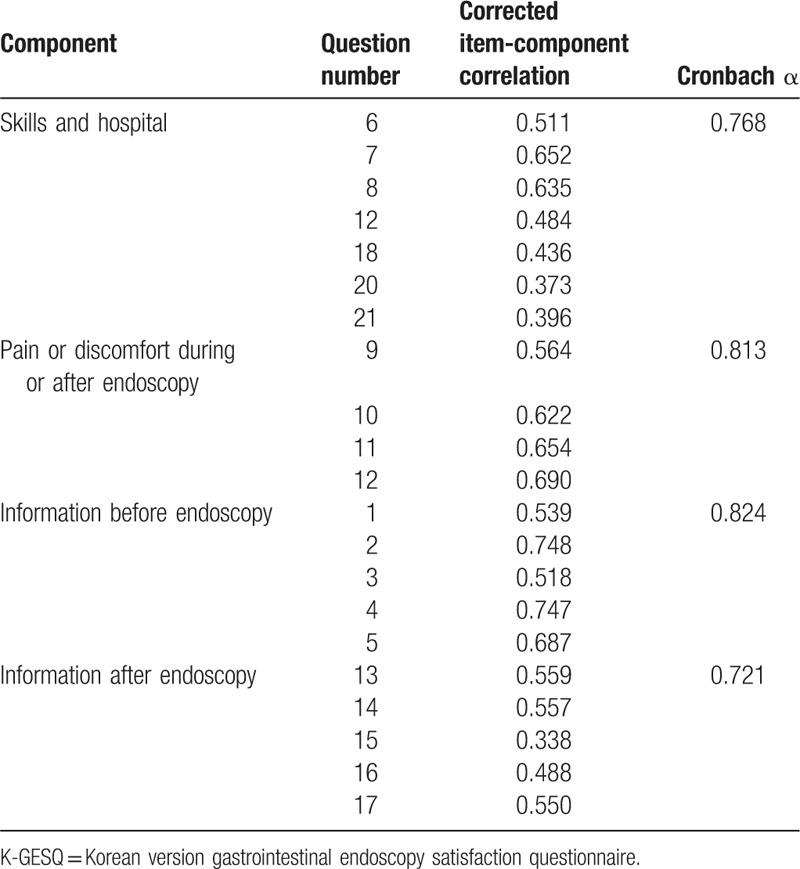
Internal consistency of the components of the K-GESQ.

### 3.4 Factors influencing the patient satisfaction measured by the K-GESQ

Influencing factors for patient satisfaction with GIE were identified through comparison with the mean K-GESQ according to various demographic factors, listed in Table 5. Female and nonsmoker were associated with less endoscopic examination satisfaction (*P* = .021 and *P* = .006, respectively). Other factors, including age, alcohol, education or economic level, sedation during endoscopy, type of endoscopy, experience with previous endoscopy, and additional costs after endoscopic examination were not identified as factors influencing endoscopy satisfaction.

**Table 5:**
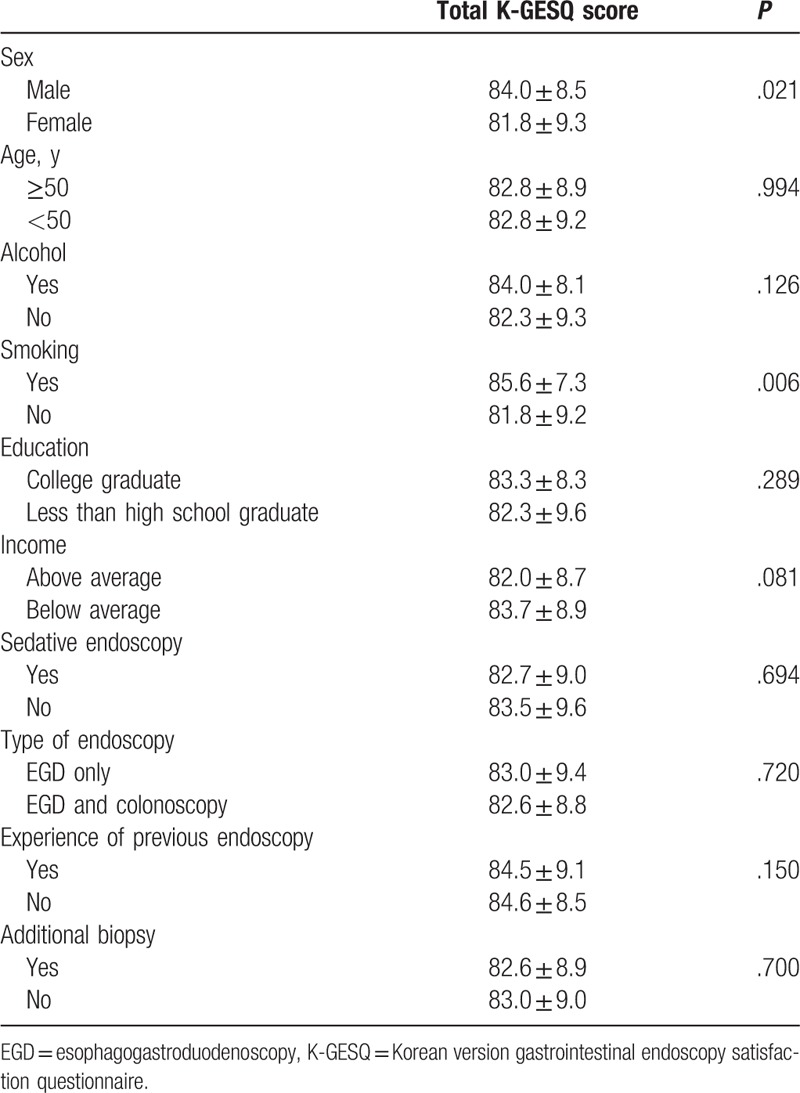
Factors influencing K-GESQ demographics.

## 4 Discussion

This study is the first validation of the GESQ in an Asian country and established its construct validity using both EFA and CFA. The correlation coefficient between the K-GESQ and a 5-point Likert scale used to test convergent validity was 0.513 in this study; as the correlation coefficient approached 0.5, K-GESQ is distinguished from a self-appointed Likert scale.^[16]^ Additionally, CFA provided evidence that the 4 domains clustered independently without multicollinearity and reflected an adequate satisfaction scale. The high value of the total Cronbach α in this study demonstrated the excellent internal consistency of the K-GESQ. The high values of all subscales consequently supported that the clustering within them was homogenous and represented the same underlying construct. As a result, this study demonstrated that the GESQ was a valid instrument for quantitative assessment of satisfaction in patients undergoing GIE in Asia.

The original GESQ was designed to reflect comprehensive patient-reported experience measures and was validated in a large multicenter endoscopic unit in the United Kingdom (UK).^[6]^ At present, the lack of consistent scales for patient satisfaction measuring the whole process in endoscopic units for patients undergoing EGD and/or colonoscopy led us to translate the recently developed GESQ.^[18]^ Most questionnaires translated from English to another language for cross-cultural utility might have the potential limitations of ethnocentricity and cultural hegemony,^[10]^ as the development of a reliable questionnaire for health care service depends on the patients who participated in the questionnaire, the medical environment, and methods applied. Nevertheless, it was shown that the GESQ was able to be applied in Korea, despite different health care utilization patterns and resources between Korea and the UK. This may be due to the similar health care systems in the 2 countries; the National Health Service (NHS) in the UK and National Health Insurance in Korea.^[19]^ In a public health care system, providers need to deliver timely medical care efficiently for universal coverage, and patients might have different desires and expectations compared to those in a private health care system. In Korea, more than 80% of all GIEs have been performed through a population-based endoscopy screening program for gastric and colorectal cancer.^[20,21]^ In this regard, the GESQ is applicable to the Korean health care system, although it was originally developed under the NHS in the UK.

Compared with the original study with a maximum 50% missing rate for some items, there was only a 1.4% missing rate for the K-GESQ items in our study. This implies the K-GESQ was comprehensible to Koreans undergoing GIE. Another advantage of the K-GESQ is its feasibility for use in daily clinical practice because it requires only 5 minutes to complete (it has only 21 items). For example, the K-GESQ is relatively shorter than the commonly used Group Health Association of America patient satisfaction survey with 60 items.^[3]^ Previous measurement tools for patient satisfaction with GIE have predominantly focused on the collection of overall satisfaction rather than the exploration of specific components related to patient perception.^[3]^ However, the GESQ contains approaches and methods used to assess endoscopy-specific and patient-derived measures.

In the original study by Hutchings et al,^[6]^ the survey was performed off-site and mailed back after GIE; consenting patients were asked to fill out the GESQ 1 day after their endoscopy and return it in a prepaid envelope. If they did not respond, they were repeatedly asked to complete a GESQ at 2 weeks and 4 weeks. As a result, the original study had a heterogeneous response time, which could affect the results, because patient satisfaction tended to decrease over time owing to recall bias, which means respondents often recalled discomfort and suffering even during the postprocedure period.^[7,22]^ Generally, there was a difference between satisfaction level from the mail-back survey off site and face to face requests on site.^[22]^ Therefore, the timing of reply from patients undergoing GIE is important. To minimize the potential for recall bias, we asked patients to complete the K-GESQ while they were still in the endoscopic center after the procedure. So, our data could be more suitable to validate the GESQ than the original data.

Our study identified that female and nonsmoker were associated with less satisfaction in patients undergoing GIE. There were 2 earlier studies showing that female played a significant role as a negative predictor of satisfaction.^[23,24]^ The possible explanations are that females are more sensitive than males to noxious stimuli, and that there are sex-based differences in the response of the human brain to somatic and gastrointestinal pain.^[25–27]^ There has been no data on the correlation between smoking and patient satisfaction with GIE. Our finding might be explained by earlier evidence that smokers had greater pain tolerance than nonsmokers because of the analgesic effect of nicotine.^[28,29]^

In contrast, the other factors (age, education or income level, endoscopist, sedative endoscopy, type of endoscopy, prior experience with endoscopy, and additional biopsy) had no impact on the K-GESQ scores in this study. Our findings were partially consistent with a previous study reporting that patient satisfaction was not different according to age, prior experience with GIE, and type of GIE.^[8]^ However, our findings were not consistent with another study, which reported that younger age, higher income, and higher educational level were associated with less satisfaction.^[23]^ In our study, sedation unexpectedly does not seem to play a significant role as a predictor of satisfaction, although endoscopy under sedation has been known to reduce anxiety and pain. This might be attributable to the need for prolonged recovery and the resulting disturbance of subsequent activities. In general, patients who are determined to receive unsedated gastroscopy and/or colonoscopy reported little difference between the pain experience during the procedure and pain or anxiety anticipated before examination.^[7]^ Thus, the purpose of comfortable endoscopy by sedation only minimally affected degree of satisfaction for those patients, which was consistent with our finding.^[30]^ Our results demonstrated that diagnostic GIE followed by abnormal findings and/or additional biopsy did not affect patient satisfaction, although this result could reflect patient feelings of loss of health.^[21]^ Several studies reported that patient satisfaction was associated with the technical skill of the endoscopist,^[7,8,21]^ which was not consistent with our findings. These discrepancies are likely due to potential variations in patients and colonoscopy factors as well as methodological differences between the different studies. Finally, our study was based on participants from a single university endoscopy center in Korea, which limits the ability to generalize our findings.

A limitation of this study is lack of demonstration for reproducibility by test–retest reliability, similar to the original GESQ study, because it was impossible for patients to undergo the same endoscopic examination twice under the same condition.^[6]^ This might cause a bias of independence among measurements with time. There was another limitation that influencing factors may not be applicable to different populations such as rural residents because this study was conducted at a single tertiary Korean endoscopy center.

Patient satisfaction is a crucial parameter that reflects quality of service associated with endoscopic practice. The questionnaire for quantitative measurement of satisfaction could help identify the specific domains of focus and document influencing factors for patient satisfaction with endoscopy. In conclusion, the K-GESQ was a valid and acceptable tool to measure patient satisfaction with GIE in Korea. Patient satisfaction measurement could contribute to systematic improvement of qualified GIE.

## Acknowledgment

The authors thank Park Ji Ae for statistical analysis.

## Author contributions

**Conceptualization:** Jae Myung Cha.

**Data curation:** Jin Young Yoon.

**Formal analysis:** Jin Young Yoon.

**Investigation:** Jin Young Yoon, Min Seob Kwak, Jung Won Jeon, Joung Il Lee.

**Methodology:** Hyun Phil Shin.

**Resources:** Min Seob Kwak, Jung Won Jeon, Hyun Phil Shin, Kwang Ro Joo.

**Supervision:** Jae Myung Cha.

**Validation:** Jin Young Yoon.

**Visualization:** Hyun Phil Shin.

**Writing – original draft:** Jin Young Yoon.

**Writing – review & editing:** Jin Young Yoon.
